# Prognostic Nutritional Index and Major Cardiovascular Events in Patients Undergoing Invasive Coronary Angiography: A Clinical Retrospective Study

**DOI:** 10.3390/jpm12101679

**Published:** 2022-10-09

**Authors:** Xiang Hu, Kanru Sang, Chen Chen, Liyou Lian, Kaijing Wang, Yaozhang Zhang, Xuedong Wang, Qi Zhou, Huihui Deng, Bo Yang

**Affiliations:** 1 Department of Endocrine and Metabolic Diseases, Key Laboratory of Clinical Laboratory Diagnosis and Translational Research of Zhejiang Province, The First Affiliated Hospital of Wenzhou Medical University, Wenzhou 325000, China; 2 School of Public Health and Management, Wenzhou Medical University, Wenzhou 325015, China; 3 Institute of Lipids Medicine, Wenzhou Medical University, Wenzhou 325015, China; 4 Department of Cardiology, the First Affiliated Hospital of Wenzhou Medical University, Wenzhou 325000, China; 5 Department of Ultrasonography, the First Affiliated Hospital of Wenzhou Medical University, Wenzhou 325000, China

**Keywords:** invasive coronary angiography, malnutrition, major cardiovascular events, prognostic nutritional index

## Abstract

We aimed to examine whether prognostic nutritional index (PNI) could serve as an auxiliary predictor for major cardiovascular events (MCEs) in patients undergoing invasive coronary angiography (ICA). A total of 485 participants were enrolled, divided into low-PNI (≥47.40) and high-PNI (<47.40) groups. ICA determined the stenotic vessels of coronary artery disease. The primary outcome was incidental MCEs, a composite of all-cause death, non-fatal myocardial infarction, non-fatal stroke, or rehospitalization of in-stent restenosis. There were 47 (9.69%) MCEs during the 3.78-years follow-up. The cumulative incidence of MCEs was significantly higher in the low-PNI patients compared with the high-PNI patients (17.07% vs. 7.18%, *p* = 0.001). Malnutrition risk (low PNI) was significantly and independently associated with a higher risk of MCEs (hazard ratios: 2.593, 95% confidence intervals [CI]: 1.418–4.742). Combined use of the number of stenotic vessels with malnutrition risk showed a higher capacity to predict the MCEs than the presence of stenotic vessels alone (areas under the receiver operator characteristic curve: 0.696 [95% CI, 0.618–0.775] vs. 0.550 [95% CI, 0.466–0.633], *p* = 0.013). In conclusion, lower PNI levels may predict a higher risk of cardiovascular events in patients undergoing ICA, which supports the necessity of the risk assessment of nutrition status and guide the clinical treatment on strengthening nutritional support before ICA is performed, as well as nutritional intervention after ICA.

## 1. Introduction

Cardiovascular disease (CVD) is one of the major contributors to global mortality, which remains the leading cause and accounts for more than 40% of deaths in China [1]. It is estimated that almost 330,000,000 individuals suffer from CVD in China, of whom more than one-third suffer from coronary artery disease (CAD) [2]. With excellent resolution and direct visualization of the coronary lumen, invasive coronary angiography (ICA) was the only method to image coronary arteries for a long time and is acknowledged indisputably as the gold standard for detecting coronary artery stenosis and determining CAD [3,4]. Despite its safe procedure, ICA still carries a small risk of major complications that results in death, stroke, or vascular dissection. Therefore, evaluating the long-term risk to life is important before ICA is performed.

Accumulating evidence supported that malnutrition could serve as a significant predictor of adverse prognosis of deaths and cardiovascular events for patients with CVD [5,6]. Nutritional status showed its priority for risk stratification because of its modifiability via health conditions and lifestyle intervention, which is accompanied by complex assessments [5,7]. The prognostic nutritional index (PNI) is a simple index comprised of serum albumin levels and lymphocyte count, reflecting the integration of nutrition, inflammation, and immunity status [8]. This index was initially generated to evaluate the immunonutritional–inflammatory status of patients undergoing gastrointestinal tract surgery [9]. Recent evidence from clinical studies has shown that decreased PNI was associated with poor long-term prognosis in patients with acute and chronic cardiovascular conditions, including acute heart failure [10], acute myocardial infarction [11], idiopathic dilated cardiomyopathy [12], and stable coronary artery disease [7].

Until recently, malnutrition risk was rarely assessed before ICA was performed. There was no evidence in a population-based retrospective study to explore the association between PNI and long-term adverse outcomes in patients undergoing ICA. Given the previous clinical findings supporting the association between malnutrition and cardiovascular development, we speculated that malnutrition was adversely associated with the incident of major cardiovascular events (MCEs) in patients who have received ICA. The present study aimed to primarily examine the association between PNI levels and the composite MCEs, including all-cause death, non-fatal myocardial infarction, and non-fatal stroke, and secondarily evaluate the clinical value of PNI on the prediction of MCEs in hospitalized patients undergoing ICA, hoping to provide the clinical evidence supporting the necessity of the assessment of nutrition status before ICA was performed.

## 2. Materials and Methods

### 2.1. Subjects

Data from a single-center, observational study of consecutive patients hospitalized in the department of cardiology, the First Affiliated Hospital of Wenzhou Medical University, from January 2016 to December 2018 were analyzed. The telephone follow-ups were conducted from May to August 2021. All study candidates underwent ICA at the time of hospitalization to determine the presence and severity of CAD (the presence and number of stenotic vessels) because they had once suffered or were just suffering chest tightness and/or chest pain. ICA was performed with a standard Judkins technique. The angiograms were interpreted by an experienced cardiologist who was unaware of the clinical information of the participants. Patients missing serum albumin levels and total lymphocyte counts (*n* = 1), and those with severe liver (*n* = 3) or renal dysfunction (*n* = 4), active inflammatory condition (*n* = 1), or previous history of malignancy (*n* = 16) were excluded from the study. In total, 485 patients were eligible for the current study, and we conducted telephone interviews with each patient, of whom 43 (8.87%) patients were missing because of the wrong telephone number (Figure S1).

The present study was carried out in accordance with the Declaration of Helsinki and approved by the Institutional Review Board of the first affiliated hospital of Wenzhou Medical University (YS2022-306). All subjects provided written informed consent before participating in the study.

### 2.2. Anthropometric Measurements

All the patients underwent a physical examination that adhered to the standard protocols by trained physicians, including anthropometric measurements (body height and weight) and blood pressure (BP). Body weight and height are measured by standard techniques and used to calculate body mass index (BMI) as follows: BMI = weight (kg)/height^2^ (m^2^). Systolic BP (SBP) and Diastolic BP (DBP) were detected using a mercury sphygmomanometer in triplicate with a 3-min interval after 10 min of rest.

### 2.3. Laboratory Measurements

Standard laboratory measurements were performed standardly as previously described [13]. In brief, all laboratory data were obtained from the first serum collection during hospitalization. The peripheral absolute value of white blood cell (WBC) (neutrophils, monocytes, and lymphocytes), fasting plasma glucose (FPG), glycated hemoglobin A1 c (HbA1 c), lipid profiles (total cholesterol [TG], triglyceride [TC], high-density lipoprotein cholesterol [HDL-c], and low-density lipoprotein cholesterol [LDL-c]), albumin (Alb), liver function (alanine aminotransferase [ALT], aspartate aminotransferase [AST]), serum creatinine (Scr), and 2 h postprandial plasma glucose (2 hPG) were measured using standard methods.

### 2.4. Definition of CVD Risk Factors and Health Conditions

The elderly were defined as individuals aged 65 years or older. Current smoking was defined as smoking ≥seven cigarettes per week for at least six months, and current drinking was defined as drinking alcohol ≥once a week in the past six months [14]. The definition of each component of metabolic status was based on the well-acknowledged criteria. In brief, overall obesity/overweight was defined as a BMI of ≥25.0 kg/m^2^ [15]; diabetes mellitus (DM) was defined as FPG of ≥7.0 mmol/L, or 2 hPG of ≥11.1 mmol/L, or HbA1 c of ≥6.5%, or a self-reported previous diagnosis of DM by healthcare professionals [16]; hypertension was defined as systolic blood pressure (SBP) of ≥140 mmHg and/or diastolic blood pressure (DBP) of ≥90 mmHg, or receiving an antihypertensive treatment [17]. Dyslipidemia was defined as serum total cholesterol (TC) level of ≥6.22 mmol/L; serum triglyceride (TG) level of ≥2.26 mmol/L; serum low-density lipoprotein cholesterol (LDL-c) level of ≥4.14 mmol/L; serum high-density lipoprotein cholesterol (HDL-c) level of <1.04 mmol/L [18]. A diagnosis of CAD was established if luminal diameter stenosis was ≥50% in more than one of the main coronary arteries [19].

### 2.5. PNI Scores for Malnutrition Risk Assessment

PNI was determined with the following formula: PNI = serum albumin (g/L) + 5 × total lymphocyte count (/nL) [9]. A PNI score ≥ 47.4 was considered a high PNI level, while a score of <47.4 was considered a low PNI level, as determined by receiver operating characteristic (ROC) curve analysis. The cut-off value of PNI < 47.4 was considered a low PNI for identifying malnutrition risk.

### 2.6. Outcome Measurements

The primary outcome was the incident MCEs, defined as a composite of all-cause death, non-fatal myocardial infarction, and non-fatal stroke, or hospitalization of in-stent restenosis that need active intervention. The incidence and onset time of MCEs were obtained by the self-reported response from the patients or their families via telephone contact. The last observation analyzed in this study was on 9 August 2021.

### 2.7. Statistical Analysis

Statistical analyses were performed with the statistical software package version 20.0 (SPSS Inc., Chicago, IL, USA). The one-sample Kolmogorov–Smirnov test accessed the normality of data distribution. The categorical data were shown as numbers and percentages. In contrast, the continuous data were shown either by mean ± standard deviation (SD) for normal-distribution data or by a median with an interquartile range of 25–75% for skewed data. The study population was divided into malnutrition (low PNI levels) and normal nutrition (high PNI levels) groups according to the cut-off value of PNI as determined by receiver operating characteristic curve (ROC) analysis. The cumulative incidence of MCEs was compared between the low PNI and high PNI groups using Kaplan–Meier analyses with a log–rank test. Cox proportional models were used to estimate the crude or multivariate-adjusted hazard ratio (HRs) with 95% confidence intervals (Cis) for the incident MCEs without or with the additional adjustments for traditional CVD risk factors, including baseline socio-demographic parameters, lifestyle factors, prevalent diseases, with the high PNI group as a reference category. Interaction tests were conducted to test whether multivariable-adjusted HRs statistically differed between the strata analyzed by including strata factors (socio-demographic factors and health status at baseline), nutritional status, and the respective interaction terms (the strata factor multiplied by nutritional status) in the Cox regression model. ROC analyses and additional areas under the ROC (AUC) analyses were carried out to evaluate the differential capacity to predict the incident MCEs between the lone presence of CAD (the presence of stenotic vessels), the severity of CAD (number of stenotic vessels), and combination of the severity of CAD with malnutrition risk (low PNI), respectively. Differences between AUCs were confirmed using the Hanley–McNeil test. Finally, sensitivity analyses, in which the patients with (1) previous history of myocardial infarction or stroke, (2) reduced left ventricular ejection fraction (<50%) in baseline, or (3) missing data of MCEs during follow-up time were separately excluded, as well as (4) the primary outcome was redefined as traditional MCE including cardiovascular death, non-fatal myocardial infarction, and non-fatal stroke (Low PNI was correspondingly redefined according to the cut-off value of 45.98 as determined by ROC with the outcome of traditional MCE), and (5) the low PNI cut-off value was set as the lowest tertile value of PNI to divide the study population, were conducted to test the statistical robustness of the final results. All *p*-values were two-tailed, and *p* < 0.05 was considered statistically significant.

## 3. Results

Four hundred eighty-five individuals receiving ICA were eligible for the study (age median 64.00 [(57.00–70.00) years). During the median follow-up period of 3.78 (3.06–4.58) years ranging from 2.42 years to 5.58 years, 47 (9.69%) MCEs occurred among the 485 participants.

### 3.1. Baseline Characteristics

Table 1 shows the difference in the socio-demographic, lifestyles, prevalent diseases, and metabolic risk factors between the high PNI and low PNI patients. There were statistical differences in the proportion of elderly population (*p* < 0.001), current smoker (*p* = 0.013), and those with overweight/obesity (*p* = 0.045) between the two groups. Regarding metabolic risk factors, only TG levels differed significantly between the high PNI and low PNI patients (*p* = 0.001).

### 3.2. Associations between Malnutrition Risk and the Incident MCEs

**Figure 1:**
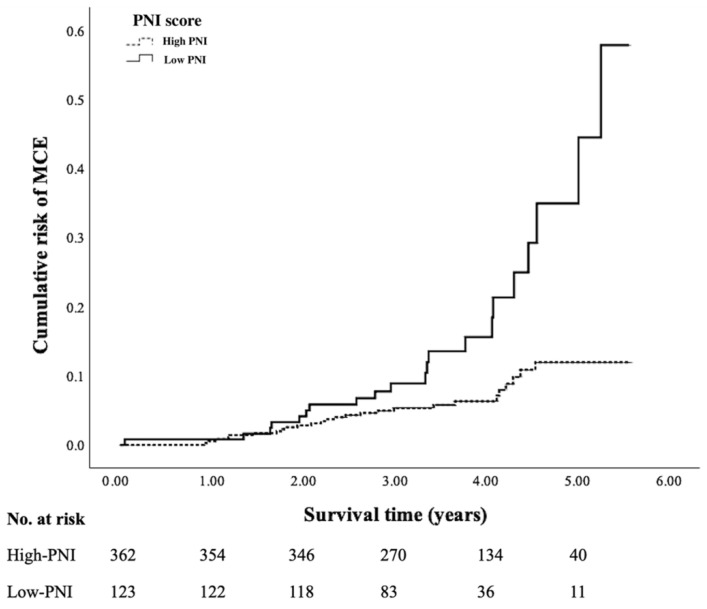
Cumulative incidence of MCEs between the low PNI and high PNI patients. Patients with low PNI scores showed a significantly higher risk of MCEs compared to those with high PNI scores (17.07%, 95% CI: 10.42–23.72% vs. 7.18%, 95% CI: 4.52–9.84%; *p* = 0.001).

Figure 1 shows the cumulative incidence of MCEs between the high PNI and low-PIN patients. Patients with low PNI levels showed significantly higher incidences of MCEs than those with high PNI levels (17.07%, 95% CI: 10.42–23.72% vs. 7.18%, 95% CI: 4.52–9.84%; *p* = 0.001).

The multivariate-adjusted HR for the association between PNI levels and the risk of MCEs is shown in Table 2. After adjustment of gender, age range, lifestyle factors, and basic health status, patients at malnutrition risk determined by low PNI levels had a higher risk of MCEs than those at high PNI levels (HR: 2.593, 95% CI: 1.418–4.742, *p* = 0.002). In sensitivity analyses separately excluding the participants with a previous history of myocardial infarction or stroke, reduced left ventricular ejection fraction (<50%) at baseline, or missing data of MCEs during the follow-up, the directions of the association between malnutrition and MCEs risk were not significantly changed (Table S1). Additionally, redefining the primary outcome as traditional MCE or the cut-off value of PNI as the lowest tertile value (<48.35) did not change the direction and significance of the association between malnutrition and long-term cardiovascular outcome (Table S1).

### 3.3. Subgroup Analyses

When stratified by socio-demographic characteristics and baseline health status (Table 3), participants with low PNI levels remained at a higher risk of incidental MCEs than those with high PNI levels in most strata analyzed. There was a significant gender difference in the association between PNI levels and risk of incidental MCEs (*p* for interaction = 0.015), with a multivariate-adjusted HR of 0.845 (95% CI: 0.314–2.273) for men and 5.055 (95% CI: 2.160–11.828) for women. There were no significant interactions of malnutrition risk with age range, overweight/obesity, and prevalent diabetes, hypertension, dyslipidemia, and CAD on the incident MCEs.

### 3.4. Receiver Operating Characteristic Analyses to Predict MCEs

**Figure 2:**
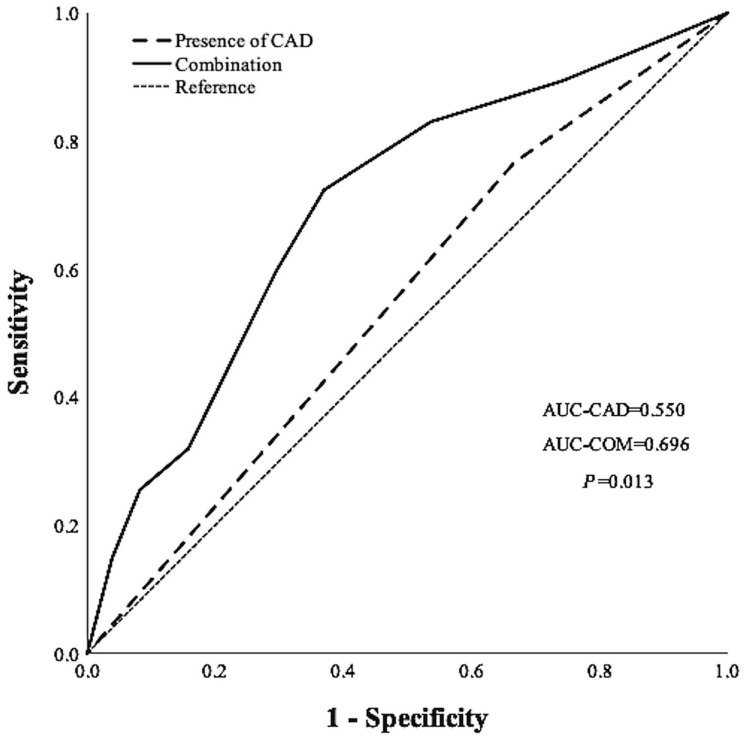
Receiver operating characteristic analyses to predict incident MCEs. Combination use of the number of stenotic vessels with prevalent malnutrition showed a significant increase in capacity to predict the incident MCEs compared to the presence of CAD alone (*p* = 0.017).

ROC analyses of the presence and severity of CAD for prediction of the MCEs had an AUC of 0.550 (95% CI, 0.466–0.633; *p* = 0.244) and 0.641 (95% CI, 0.550–0.731; *p* = 0.002), respectively, but their AUC values were not statistically different (*p* = 0.148). However, the combination of the severity of CAD with malnutrition risk had a higher AUC of 0.696 (95% CI, 0.618–0.775; *p* < 0.001) than the presence of CAD alone (*p* = 0.013) (Figure 2).

## 4. Discussion

The present study was the first clinical retrospective study supporting the association between malnutrition determined by low PNI levels and incident MCEs in patients undergoing ICA. Our major finding indicated that a lower PNI was associated with a higher risk of MCEs in patients undergoing ICA, independent of socio-demographic factors, lifestyle risk factors, and prevalent cardiometabolic status, including CAD. The malnutrition risk identified by low PNI scores strengthens the capacity of the number of stenotic vessels detected by ICA to predict poor cardiovascular prognosis. Such evidence helps fill the gap that lacks detailed associations between PNI and the risk of MCEs in patients undergoing ICA. It further supports the necessity of risk assessment of nutrition status before ICA is performed and guides the clinical treatment on strengthening nutritional support before ICA and nutritional intervention after ICA.

Evidence supports the association between lower PNI and increased risk of adverse outcomes in patients with different cardiovascular conditions. PNI was reported to be negatively and independently associated with the incidence of all-cause and cardiovascular mortality in patients with heart failure [10]. Later clinical studies conducted in patients with idiopathic dilated cardiomyopathy showed that PNI was an independent risk factor for short-term adverse events (in-hospital MCEs) and long-term all-cause deaths [12]. In terms of CAD, clinical findings have come from studies among patients with acute and stable conditions. However, they have not been consistent. For patients with stable CAD, Japanese researchers found lower PNI was associated with a higher risk of MCEs [7]. Chinese researchers reported lower PNI was associated with in-hospital and follow-up mortality rates in patients with acute myocardial infarction undergoing primary percutaneous coronary intervention [20]. Similarly, a study from Korea found that in patients with acute myocardial infarction, who were followed up for at least 1 year, the cumulative incidence of MCEs was significantly higher in patients with severe malnutrition compared to those with normal nutritional status and those with mild to moderate malnutrition. However, this study showed that lower PNI was not associated with a higher risk of all-cause deaths [21]. Kalyoncuoğlu M et al. from Turkey did not find an association between PNI and incidence of one-year major adverse cardiac and cerebrovascular events. Similarly, Basta G et al. from Italy found no association between PNI and long-term all-cause deaths in patients with myocardial infarction [22,23].

Consistent with most previous studies, the present study discovered that for patients undergoing ICA who have suffered angina and are highly suspected of having CAD, a lower PNI is associated with a higher risk of long-term MCEs. Additionally, low PNI was an independent risk factor for poor cardiovascular outcomes. This finding suggested that PNI might be a simple and effective indicator for the risk stratification of patients before the ICA was performed. Furthermore, an evaluation of nutritional status is recommended in the American and European guidelines for patients with heart failure [24,25]. However, the importance of nutritional assessment to patients with CAD has been poorly considered, especially for those undergoing ICA before the diagnosis of CAD. Therefore, this finding provides clinical evidence supporting the need to assess nutrition status using PNI and initiate nutritional intervention before and after ICA.

The results of our subgroup analysis found that women and the elderly were more vulnerable to malnutrition. Data from multicenter studies in acute care settings showed that 23−60% of elderly patients were malnourished, and approximately a quartile of elderly patients was at risk of malnutrition [26]. Chinese researchers reported that in elderly patients with CVD, there were a higher proportion of female patients with malnutrition or a higher risk of malnutrition than men [27]. Malnutrition contributes to a higher risk of CVD and adverse outcomes, such as aggravated malnutrition. Therefore, women and the elderly, who are at higher risk of malnutrition, were more predisposed to the negative outcome of malnutrition [26]. The present study also supported strengthening the malnutrition-adverse CVD outcome loop in women and the elderly, showing that the association between malnutrition and MCEs was more pronounced in women and the elderly. Additionally, underlying chronic diseases were often related to malnutrition via the direct impact on nutritional intake and other metabolic and/or psychological disorders, which might further strengthen the long-term influence of malnutrition on cardiovascular outcomes [28,29]. In consideration of the potential influence of underlying chronic metabolic-cardiovascular diseases, the present study, however, discovered that the baseline chronic diseases, including overweight/obesity, diabetes, hypertension, dyslipidemia, and even CAD, did not interact with the association of PNI and the incidence of MCEs. Hence, PNI was appropriate to serve as an indicator for MCEs risk in patients, especially women and the elderly, with different metabolic-cardiovascular risks who have received ICA.

ICA was traditionally used to investigate and describe the coronary anatomy of patients with stable chest pain suspected to be caused by CAD. However, there is a discrepancy between angiographic severity and myocardial ischemia [30]. In addition, several thousand patients suffer adverse events from ICA annually [31]. Our ROC analyses suggested the importance of nutritional assessment before the performance of ICA, not only for strengthening the capacity of angiographic severity detected by ICA to predict the long-time cardiovascular outcome but also for the risk stratification and reference for the following appropriate management plan.

The mechanism underlying the association between PNI and adverse cardiovascular outcomes remained unclear. Decreased PNI reflects the imbalance and deficiency of immunonutritional–inflammatory status. Since PNI was comprised of serum albumin levels and lymphocyte count, the potential mechanism could be explained in the aspect of albumin and lymphocytes, respectively. A lower circulating albumin level is known to be associated with inflammation that contributes to the development of MCEs. The lower circulating albumin levels reflected increased vascular permeability in inflammatory processes [32]. In addition, as an antioxidant, albumin can scavenge free reactive oxygen and nitrogen species and bind eicosanoids and nitric oxide. A decrease in albumin attenuates its function to improve endothelial dysfunction, regulate vascular tone, suppress platelet aggregation, and maintain endothelial permeability [33,34]. Immune cells, especially lymphocytes, are susceptible to nutrient deprivation and decrease rapidly in malnutrition. The decreased lymphocytes with limited activity cannot initiate a successful immune response, resulting in a poor outcome [35]. The reduction in the proportion of lymphocytes indicated the physiological stress response. This reflects the cascade reactions of the activation of the hypothalamic-pituitary-adrenal axis, which are correlated to the systemic response to the deteriorating cardiovascular system [36].

There are also several limitations of this study. First, the present study was a single-center study with a relatively small sample size. This limited the power of the statistical test in revealing significant effects of PNI on MCEs in different subgroups. Second, we did not extend the inclusion period beyond December 2018, which would likely result in a limited number of eligible participants. Further studies should be conducted to extend the inclusion period to generalize and confirm the present finding. Third, PNI levels were evaluated only once but not assessed again over time during the follow-up period. Therefore, we cannot determine whether the improvement or decline in PNI would alter the long-term prognosis. Forth, the onset and occurrence time of MCEs were self-reported by the patients or their families, which may result in potential recall bias. Fifth, variables associated with malnutrition and frailty, such as weight loss, grip strength, or muscle wasting, were not involved in the present study. The lack of these malnutrition-related factors might explain the attenuated association between low PNI and MCEs risk in patients with diabetes because of the complex nutritional assessment for patients with diabetes, which should consider the balance between strict diet for glucose control and increased risk of frailty and sarcopenia related to diabetes and malnutrition. The association of PNI and MCEs should be further investigated, considering these confounding factors. Lastly, the sample size was relatively small, especially in the sub-groups of those without hypertension or dyslipidemia, and the follow-up time was relatively too short to observe the primary outcome.

## 5. Conclusions

The present study suggests that malnutrition risk identified by lower-PNI scores contributes to a higher risk of long-term cardiovascular events in Chinese patients undergoing ICA. PNI might be a simple and effective indicator for cardiometabolic risk stratification and should be considered before cardiovascular-related interventions are performed. Further studies are needed to confirm and elucidate the findings in larger cohorts with a more extended follow-up period to validate the clinical application of PNI in managing cardiovascular-related interventions.

## Figures and Tables

**Table 1:** Baseline Characteristics of the study population.

Variable	Total (*n* = 485)	High PNI (*n* = 362)	Low PNI (*n* = 123)	*p*
***Socio-demographics factors***				
Men (n, %)	253 (52.16%)	188 (51.93%)	65 (52.85%)	0.861
Elderly (n, %)	235 (48.45%)	158 (43.65%)	77 (62.60%)	<0.001
***Lifestyle risk factors***				
Current smoking (n, %)	92 (18.97%)	78 (21.55%)	14 (11.38%)	0.013
Current drinking (n, %)	21 (4.33%)	19 (5.25%)	2 (1.63%)	0.088
***Baseline health status***				
Overall overweight/obesity (n, %)	301 (62.06%)	234 (64.64%)	67 (54.47%)	0.045
Diabetes (n, %)	279 (57.53%)	202 (55.80%)	77 (62.60%)	0.187
Hypertension (n, %)	388 (80.00%)	292 (80.66%)	96 (78.05%)	0.531
Dyslipidemia (n, %)	390 (80.41%)	293 (80.94%)	97 (78.86%)	0.616
Coronary artery disease (n, %)	328 (67.63%)	244 (67.40%)	84 (68.29%)	0.855
***Metabolic risk factor***				
BMI (kg/m^2^)	26.08 (24.13–28.06)	26.11 (24.29–28.08)	25.96 (23.51–28.08)	0.467
SBP (mmHg)	138.00 (124.00–152.50)	138.00 (124.00–152.00)	138.00 (124.00–154.00)	0.953
DBP (mmHg)	77.00 (68.50–84.00)	77.00 (69.00–84.25)	78.00 (67.00–83.00)	0.837
FPG (mmol/L)	5.40 (4.70–6.80)	5.40 (4.70–6.80)	5.20 (4.68–6.82)	0.483
2 hPG (mmol/L)	12.133 ± 3.76	12.09 ± 3.94	12.28 ± 3.21	0.774
HbA1 c (%)	6.30 (5.80–7.48)	6.30 (5.90–7.20)	6.30 (5.60–7.80)	0.642
TG (mmol/L)	1.88 (1.41–2.54)	1.94 (1.49–2.61)	1.64 (1.23–2.29)	0.001
TC (mmol/L)	4.47 (3.78–5.34)	4.51 (3.85–5.33)	4.31 (3.63–5.36)	0.402
HDL-c (mmol/L)	0.98 (0.82–1.14)	0.99 (0.84–1.13)	0.94 (0.80–1.16)	0.158
LDL-c (mmol/L)	2.49 (1.91–3.20)	2.51 (1.94–3.18)	2.35 (1.78–3.23)	0.381

Abbreviation: MCE: major cardiovascular events; BMI: body mass index; SBP: systolic blood pressure; DBP: diastolic blood pressure; FPG: fasting plasma glucose; 2 hPG: 2-h plasma glucose; HbA1 c: glycated hemoglobin A1 c; TG: triglyceride; TC: total cholesterol; HDL-c: high-density lipoprotein cholesterol; LDL-c: low-density lipoprotein cholesterol; PNI: prognostic nutritional index. Definition: Current smoking: smoking ≥seven cigarettes/week for at least six months; Current drinking: drinking alcohol ≥once a week in the past six months; Overall obesity/overweight: defined as a BMI of ≥25.0 kg/m^2^; Diabetes mellitus: FPG of ≥7.0 mmol/L, or 2 hPG of ≥11.1 mmol/L, or HbA1 c of ≥6.5%; Hypertension: systolic blood pressure of ≥140 mmHg and/or diastolic blood pressure of ≥90 mmHg; Dyslipidemia: serum total cholesterol level of ≥6.22 mmol/L; serum triglyceride level of ≥2.26 mmol/L; serum low-density lipoprotein cholesterol level of ≥4.14 mmol/L; serum high-density lipoprotein cholesterol level of <1.04 mmol/L; Coronary artery disease: luminal diameter stenosis was ≥50% in more than one of the main coronary arteries.

**Table 2:** Association between the prognostic nutritional index levels and major cardiovascular events risk.

PNI Scores (Low vs. High)	Hazard Ratios	95% Confidence Intervals	*p*
Model 1	2.579	1.450–4.590	0.001
Model 2	2.406	1.334–4.337	0.004
Model 3	2.592	1.426–4.711	0.002
Model 4	2.593	1.418–4.742	0.002

Model 1: Unadjusted; Model 2: Adjusted for gender, age range; Model 3: Adjusted for gender, age range, and lifestyle risk factors (current smoking, current drinking); Model 4: Adjusted for gender, age range, lifestyle risk factors, and baseline health status (overweight/obesity, diabetes, hypertension, dyslipidemia, coronary artery disease).

**Table 3:** Interactions of subgroup factors on the association between prognostic nutritional index levels and major cardiovascular events risk.

Variable	Total (N)	MCE (N)	MCE (%)	Hazard Ratios	95% Confidence Intervals	*p*	*p* for Interaction
***Socio-demographics factors***							
Gender							0.015
Men	253	24	9.49	0.845	0.314–2.273	0.739	
Women	232	23	9.91	5.055	2.160–11.828	<0.001	
Age range							0.051
<65	250	18	7.20	0.808	0.225–2.904	0.744	
≥65	235	29	12.34	4.202	1.920–9.198	<0.001	
***Baseline health status***							
Overall overweight/Obesity							0.604
Yes	301	30	9.97	2.842	1.318–6.129	0.008	
No	184	17	9.24	2.811	0.987–8.006	0.053	
Diabetes							0.376
Yes	279	30	10.75	1.810	0.804–4.076	0.152	
No	206	17	8.25	4.195	1.514–11.623	0.006	
Hypertension							0.429
Yes	388	41	10.57	2.860	1.488–5.498	0.002	
No	97	6	6.19	1.126	0.164–7.746	0.904	
Dyslipidemia							0.384
Yes	390	34	8.72	2.253	1.087–4.669	0.029	
No	95	13	13.68	4.520	1.432–14.272	0.010	
Coronary heart disease							0.314
Yes	328	36	10.98	2.102	1.034–4.272	0.040	
No	157	11	7.01	4.406	1.174–16.532	0.028	

Definition: Current smoking: smoking ≥seven cigarettes/week for at least six months; Current drinking: drinking alcohol ≥ once a week in the past six months; Overall obesity/overweight: defined as a BMI of ≥25.0 kg/m^2^; Diabetes mellitus: FPG of ≥7.0 mmol/L, or 2 hPG of ≥11.1 mmol/L, or HbA1 c of ≥6.5%; Hypertension: systolic blood pressure of ≥140 mmHg and/or diastolic blood pressure of ≥90 mmHg; Dyslipidemia: serum total cholesterol level of ≥6.22 mmol/L; serum triglyceride level of ≥2.26 mmol/L; serum low-density lipoprotein cholesterol level of ≥4.14 mmol/L; serum high-density lipoprotein cholesterol level of <1.04 mmol/L; Coronary artery disease: luminal diameter stenosis was ≥50% in more than one of the main coronary arteries.

## Data Availability

The data presented in this study are available on request from the corresponding author. The data are not publicly available due to ethical reasons.

## Supplementary Materials

The following supporting information can be downloaded at: https://www.mdpi.com/article/10.3390/jpm12101679/s1, Figure S1. Flow diagram of the study process; Table S1. Sensitivity analyses of the association between malnutrition and major cardiovascular event risk.
