# Supplementary material for: Prognostic Nutritional Index and Major Cardiovascular Events in Patients Undergoing Invasive Coronary Angiography: A Clinical Retrospective Study
Source: J Pers Med. 2022 Oct 9;12(10):1679. doi: 10.3390/jpm12101679 (PMC9604840; doi:10.3390/jpm12101679)
Supplement: Supplementary file 1 [file jpm-12-01679-s001.zip › jpm-1797120-supplementary.pdf]

Table S1 Sensitivity analysis of the association between the prognostic nutritional index levels and major cardiovascular events risk

	Participants n	MCE case (n, %)	Hazard Ratios	95% Confidence Intervals	***P***
**Excluding previously incidence of myocardial infarction or stroke**					
Crude model	416	35 (8.41%)	2.748	1.410–5.355	0.003
Multi-adjusted model			2.659	1.289–5.484	0.008
**Excluding reduced left-ventricular ejection fraction (< 50%) in baseline**					
Crude model	462	42 (9.09%)	2.680	1.452–4.945	0.002
Multi-adjusted model			2.704	1.420–5.147	0.002
**Excluding missing data of MACE during the follow-up**					
Crude model	442	46 (10.41%)	2.614	1.462–4.672	0.001
Multi-adjusted model			2.734	1.487–5.026	0.001
**Redefining the primary outcome of traditional MCE**					
Crude model	485	41 (8.45%)	2.934	1.552–5.546	0.001
Multi-adjusted model			2.991	1.538–5.818	0.001
**Setting the low PNI cut-off as the lowest-tertile value (< 48.35)**					
Crude model	485	47(9.69%))	1.981	1.118–3.510	0.019
Multi-adjusted model			1.947	1.071–3.538	0.029

Abbreviations: MCE: major cardiovascular events; PNI: prognostic nutritional index.

Traditional MCE included cardiovascular death, non-fatal myocardial infarction, and non-fatal stroke; Low PNI was correspondingly redefined according to the cut-off value of 45.98, as determined by the receiver operating characteristic curve with the outcome of traditional MCE.

Crude model: Unadjusted.

Multi-adjusted model: Adjusted for gender, age range, lifestyle risk factors (currently smoking, currently drinking), and baseline health status (overweight/obesity, diabetes, hypertension, dyslipidemia, and coronary artery disease).
