# Supplementary figures and images for: Prognostic Nutritional Index and Major Cardiovascular Events in Patients Undergoing Invasive Coronary Angiography: A Clinical Retrospective Study
Source: J Pers Med. 2022 Oct 9;12(10):1679. doi: 10.3390/jpm12101679 (PMC9604840; doi:10.3390/jpm12101679)

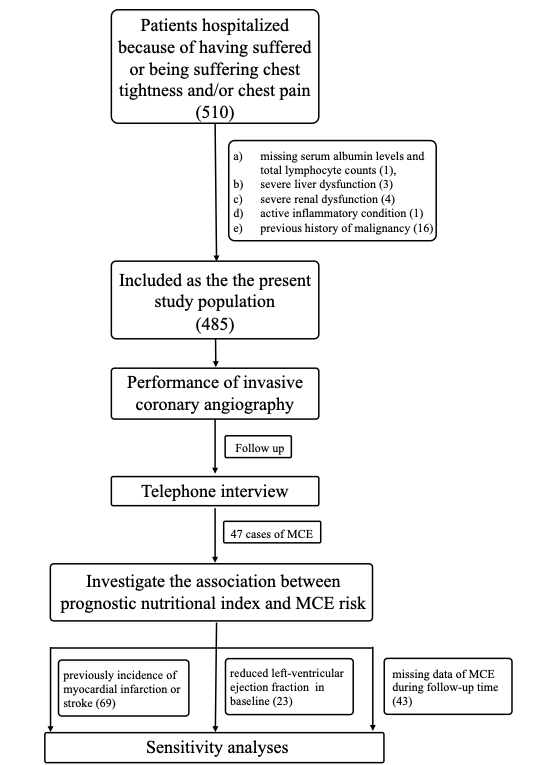

Supplement: Supplementary file 1 [file jpm-12-01679-s001.zip › Figure S1.tiff]
